# Dietary Effects on Cuticular Hydrocarbons and Sexual Attractiveness in *Drosophila*

**DOI:** 10.1371/journal.pone.0049799

**Published:** 2012-12-05

**Authors:** Tatyana Y. Fedina, Tsung-Han Kuo, Klaus Dreisewerd, Herman A. Dierick, Joanne Y. Yew, Scott D. Pletcher

**Affiliations:** 1 Department of Molecular and Integrative Physiology, University of Michigan, Ann Arbor, Michigan, United States of America; 2 Department of Molecular and Human Genetics, Baylor College of Medicine, Houston, Texas, United States of America; 3 Institute of Medical Physics and Biophysics, University of Münster, Münster, Germany; 4 Department of Biological Sciences, National University of Singapore, Singapore, Singapore; 5 Temasek Life Sciences Laboratory, Singapore, Singapore; 6 Geriatrics Center, University of Michigan, Ann Arbor, Michigan, United States of America; University of Arkansas, United States of America

## Abstract

Dietary composition is known to have profound effects on many aspects of animal physiology, including lifespan, general health, and reproductive potential. We have previously shown that aging and insulin signaling significantly influence the composition and sexual attractiveness of *Drosophila melanogaster* female cuticular hydrocarbons (CHCs), some of which are known to be sex pheromones. Because diet is intimately linked to aging and to the activity of nutrient-sensing pathways, we asked how diet affects female CHCs and attractiveness. Here we report consistent and significant effects of diet composition on female CHC profiles across ages, with dietary yeast and sugar driving CHC changes in opposite directions. Surprisingly, however, we found no evidence that these changes affect female attractiveness. Multivariate comparisons among responses of CHC profiles to diet, aging, and insulin signaling suggest that diet may alter the levels of some CHCs in a way that results in profiles that are more attractive while simultaneously altering other CHCs in a way that makes them less attractive. For example, changes in short-chain CHCs induced by a high-yeast diet phenocopy changes caused by aging and by decreased insulin signaling, both of which result in less attractive females. On the other hand, changes in long-chain CHCs in response to the same diet result in levels that are comparable to those observed in attractive young females and females with increased insulin signaling. The effects of a high-sugar diet tend in the opposite direction, as levels of short-chain CHCs resemble those in attractive females with increased insulin signaling and changes in long-chain CHCs are similar to those caused by decreased insulin signaling. Together, these data suggest that diet-dependent changes in female CHCs may be sending conflicting messages to males.

## Introduction

Sexual attractiveness and individual fitness are intimately linked as animals evolve to recognize the traits that indicate high fitness and reproductive potential in their potential mates [1], [2]. Diet is one of the primary environmental factors that influences fitness, and it has long been known that dietary restriction without malnutrition affects lifespan and reproductive output across a wide range of organisms from nematode worms to mammals [3]. Emerging evidence suggests that it is diet composition, such as the ratio of protein to carbohydrate, rather than caloric content *per se,* that is responsible for the observed effects [4], [5], [6]. This may not be surprising as various physiological and sex-specific tasks require particular nutrients, and certain allocation decisions, such as those directed towards survival versus reproduction, are often optimized by different diets [7], [8], [9], [10]. Because diet composition can vary widely over an individual’s lifetime, natural selection is likely to favor biological mechanisms that rapidly alter allocation decisions in response to nutrient availability as well as mechanisms in individuals of the opposite sex to evaluate such decisions in their potential mates.

Many insects assess reproductive value of potential mates based on the chemical signature of cuticular hydrocarbons (CHCs), which are long-chain lipids synthesized from fatty acid precursors and deposited on the insect cuticle. Their presumed ancestral function is desiccation resistance, but they also play a major role in insect social communication and recognition of species, sex, dominance, and reproductive status [11]. CHC composition has been shown to respond to diet and other environmental changes in a variety of insects [10], [12], [13], suggesting interactions among major metabolic pathways and CHC biosynthesis. In *D. melanogaster*, a few of the cuticular hydrocarbons, namely female-specific 7,11-heptacosadiene (7,11-HD) and 7,11-nonacosadiene (7,11-ND), have been shown to act as sex pheromones [14], with 7,11-HD being the most abundant CHC in non-African strains and also the most potent inducer of male courtship [15]. Among the remaining thirty-plus identified CHCs, some have been suggested to exhibit small additive effects on sexual attractiveness [16], but most have unidentified functions. Very long-chain CHCs (C31–35) are abundant in immature adult flies, when both sexes are vigorously courted by mature males, but these CHCs greatly diminish within a day after eclosion [17].

Recent studies have shown that CHC composition and sexual attractiveness in *Drosophila melanogaster* are strongly influenced by aging [18] as well as by genetic manipulations of the insulin-insulin-like signaling (IIS) and target-of-rapamycin (TOR) pathways [19]. CHCs of young females and of females with increased IIS are more attractive to males than CHCs of old females or of females with decreased IIS [18], [19]. Aging and IIS are both strongly affected by diet [20], but how CHCs are modified by diet composition and whether any observed changes will impact the attractiveness of female pheromone profiles are unclear. While, in general, increased dietary sugar is thought to promote IIS [21] and increased dietary protein putatively activates the TOR pathway [22], these two major nutrient-sensing pathways are known to interact [23], [24], making it difficult to predict a resulting phenotype when the activity of both pathways might be altered. For instance, dietary yeast and sugar produce opposing effects on a number of physiological traits in *Drosophila*, including fecundity, triglyceride levels, sleep, and activity patterns, while having similar effects on others, such as longevity [25], [26]. Female fecundity, in particular, is known to be compromised by aging, reduced insulin signaling, and yeast restriction [26], [27]. Any one or more of these traits may serve as indicators of individual fitness and therefore may contribute to mate choice.

This study set out to test whether dietary protein and sugar affect CHC composition and female attractiveness across different ages. Based on the above considerations, we predicted that increasing dietary yeast and sugar would have significant but opposing effects on these phenotypes. Under the assumption that males would prefer females with higher immediate reproductive potential, we further anticipated that females fed high-yeast diets would be more attractive because they are more fecund [26]. We find that CHCs are differentially affected by increases in dietary sugar and yeast, and that the differences are exacerbated by age. Surprisingly, however, females fed different diets showed no detectable difference in sexual attractiveness at any age. We discuss potential explanations for these results and draw parallels among the effects of diet, age, and insulin signaling on female CHCs and attractiveness.

## Materials and Methods

### Fly Stocks and Husbandry

Canton-S flies were obtained from the Bloomington Stock Center. For all experiments, larvae were cultured in cornmeal-sugar-yeast “larval” media. Virgin adults were collected shortly after eclosion and supplied with fresh S10Y10 food (represented in terms of sugar%yeast%) every 2–3 days. A balanced diet in the range of S5Y5 to S10Y10 has been determined to maximize longevity of *D. melanogaster* females in the laboratory [26]. S5Y5 diet was, therefore, chosen as a base (control) diet for this study, while S20Y5, S5Y20, and S20Y20 represented diets with increased sugar, yeast, or both, respectively. These diets were chosen based on extensive characterization of their effects on fly lifespan and physiology [26]. Notably, yeast is a major source of protein. However, it also provides the dietary source of other nutrients including lipids, fatty acids, and vitamins [28]. Amino acid supplementation alone has been shown to reverse the effects of a low-yeast diet on lifespan and reproductive output; micronutrients and lipids had little effect [5], [29]. On the other hand, even though flies are able to synthesize all the necessary fatty acids *de novo*, they do effectively incorporate dietary sources of fatty acids into a variety of tissues [30], making it possible that dietary yeast may impact CHC production in a variety of ways. All flies were maintained at 25°C and 60% relative humidity in a 12∶12 h light:dark cycle. Details concerning media recipes can be found in [26]. We chose to study female CHCs because the effects of diet, age, and IIS are larger in magnitude and better understood mechanistically in this sex [18], [19], [31]. Furthermore, female fecundity is more tightly linked to diet than male sexual performance in a single mating event [26], [32], [33], [34], suggesting that mate choice based on current nutritional status is an especially advantageous strategy for males.

### Cuticular Hydrocarbon Analysis

CHCs were analyzed by gas chromatography mass spectrometry (GC-MS) and by laser desorption/ionization orthogonal time-of-flight mass spectrometry (LDI-MS) as described in more detail in [18], [19]. GC-MS analysis provides quantification of individual alkanes, alkenes, and branched hydrocarbon species while LDI-MS is better suited for analysis of more polar lipid components such as hydrocarbons with oxygen functional groups. LDI-MS does not detect alkanes. For GC-MS analysis, a large cohort of flies was established by placing same-age virgin females into 95×25 mm^2^ vials containing 10 ml of one of 4 different media: S5Y5, S20Y20, S20Y5 and S5Y20. Three replicates of 5 flies were sampled at 6, 24, 47 and 61 days of adult age (i.e., days since eclosion). Each sample of 5 flies from a single vial was placed in 100 µl of hexane, which contained 10 µg/ml of hexacosane (Sigma-Aldrich) as an internal standard. Following incubation at room temperature (23°C) for 30 min, the cuticular extract was removed and placed in a clean glass vial. The solvent was then evaporated under a chemical hood for 1–2 h, and the dry extracts were stored at −80°C. After all samples had been collected, each one was re-dissolved in 30 µl of heptane and analyzed by GC-MS analysis. All samples were processed on the same day. The GC-MS analysis was performed as described in [18], [19]. Compounds were identified on the basis of retention time and electron ionization (EI) mass spectra. The signal intensity of each compound was calculated as the area under its corresponding peak. Relative CHC profiles were derived by dividing individual peak intensities by the sum of the intensities of all identified hydrocarbons. To obtain a measure for the total amount of CHC, the sum of the signal intensities of all identified compounds was divided by that of the internal standard.

For LDI-MS, multiple independent cohorts were established every 2–3 weeks. Virgin females were sorted onto the four different food media and were sampled on the same day for CHC analysis (described in more detail in [18]). Flies were anesthetized and mounted with fine forceps onto adhesive tape. Mass-spectra were acquired from both the foreleg tarsi and ano-genital regions. These two measures were highly correlated [18], and we chose to focus on the tarsal data for further analysis. The orthogonal mass spectrometer was equipped with an N_2_ laser emitting 3 ns long pulses at a wavelength of 337 nm and a repetition rate of 30 Hz. The laser beam spot size on the sample is ∼200 µm in diameter and has an approximately flat-top intensity profile. Ions were generated in a buffer gas environment using 2 mbar of Argon gas. For acquisition of a mass spectrum, 900 laser pulses were applied to one spot for 30 s. Laser fluence (light energy per pulse and area) was adjusted to values moderately above the ion detection threshold, corresponding to values between 100–200 J/m^2^. All data were acquired in positive ion mode, and mass spectra were processed using the MoverZ software (v. 2001.02.13, Genomic Solutions). Potassiated molecules [M+K]^+^ formed the dominant hydrocarbon ion signals in all LDI-MS mass spectra. Elemental composition assignments are based on the assumption that the observed and theoretical mass values agree within +/−0.02 Da. Relative quantification was expressed using a normalized intensity (also referred to as relative abundance, relative amount), derived by dividing individual signal intensities by the sum of the intensities of all identified hydrocarbon signals.

### Behavioral Assay

Female attractiveness was tested using a two-choice behavioral assay in which flies were video recorded and then analyzed using fly tracking software [18]. In this assay, two subject females were decapitated and embedded (legs only) in agar 15–20 mm apart and 7–10 mm away from the side of the dish. After the agar solidified, a single, 4–8 day-old virgin Canton-S male was released in the arena and given 10 min to acclimate to the new environment. Video recording was then started and continued for 30 min. Videos were recorded at 2 frames per second and converted to AVI file format, which was analyzed with VideoFly software that was developed in our laboratory. The software was written in C and C# and was built around the OpenCV image analysis library (http://opencv.willowgarage.com/wiki/); it is freely available from our laboratory website (http://sitemaker.umich.edu/pletcherlab/). VideoFly calculates the amount of time spent by a focal fly inside a circle of 3 mm radius centered on each decapitated subject fly. Instances where the total time spent inside a defined region was less than 50 s were removed from further analysis to standardize the denominator for proportional data. As with our courtship assay, male preference was calculated as the percentage of time males spent in the circles centered on one of the subject females divided by the total time spent in both.

### Statistical Analysis

To visualize and interpret general changes in CHC profiles with age and diet, principal component analysis (PCA [35]) was used for the two datasets obtained by GC-MS (26 CHCs) and LDI-MS (12 CHCs). To increase the power of PCA, previous data on CHC changes with age in two fly strains (a standard laboratory Canton-S strain and a recently wild-caught strain called Fv [18]) on a balanced S10Y10 diet were also used to extract principal components (PCs). This resulted in 78 total CHC samples for GC-MS dataset and 135 samples for LDI-MS dataset. PCA was performed on the correlation matrix; three PCs were retained; and Varimax rotation was applied to improve interpretability of component loadings. Because LDI-MS analysis was performed on individual flies, it resulted in much tighter correlation among individual CHCs compared to GC-MS data (where 1 sample = CHCs of 5 flies). Consequently, ANOVA on principal components scores from LDI-MS dataset was used to test the effects of diet, age, and their interaction on each principal component (PC).

For individual CHCs, two-factor ANOVAs were applied to test the effects of diet, age, and their interaction, with transformation applied when required to conform the data to a normal distribution. Results of behavioral trials, expressed as a mate preference (i.e., the percentage time a male spent in the courtship area of one of the two females over total courtship time directed to both females), were analyzed using Wilcoxon signed rank test for difference from 50% ( = no choice). All analyses were performed in JMP 9 (SAS Institute Inc). A sequential Bonferroni correction was applied when assessing the impact of high dietary sugar versus high yeast on individual CHC, and differences were considered significant in the context of an experiment-wise α <0.05.

Qualitative meta-analysis comparing directions of changes in individual CHCs in response to age, diet and insulin signaling was done using the data from this study and from our two previous studies [18], [19]. When two or more datasets were used to derive P-values for CHC response to the same factor, direction and statistical significance of change in each individual CHC was tabulated based on the prevalent direction and significance.

## Results and Discussion

### Global Responses of CHC Profiles to Diet

**Figure 1:**
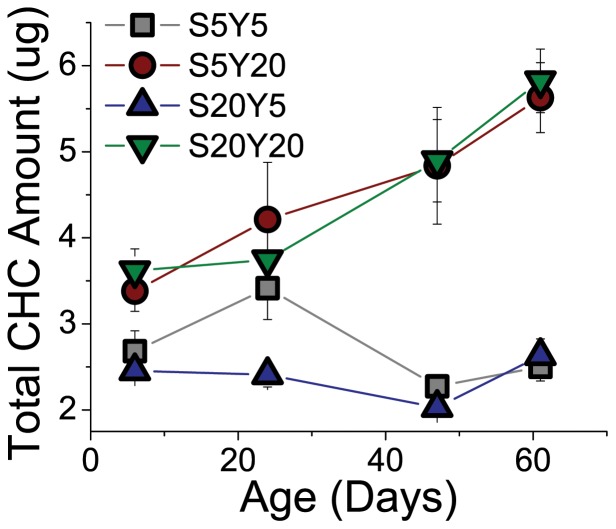
Total amounts of CHCs determined by GC-MS analysis for females *D. melanogaster* maintained on different diets. ANOVA on log-transformed data yielded highly significant effects of diet (P<0.0001), age (P = 0.0038), and diet by age interaction (P = 0.0003) on total amount of CHC. Based on Tukey HSD post-hoc tests total amounts of CHC in S5Y5 and S20Y5 females are not different from each other and are both different from S5Y20 and S20Y20 females. This indicates that protein in the diet is the main determinant of the total CHC amount.

The total amount of CHCs detected by GC-MS was affected strongly by diet. While flies from all diets exhibited similar levels of CHCs early in life, there was a marked (nearly two-fold) increase in total CHCs on high-yeast diets with age, irrespective of the amount of dietary sugar (Fig. 1). Flies fed low-yeast diets maintained roughly equivalent levels of CHC throughout their life. The amount of dietary sugar had no effect on total CHC levels or their change with age. One possible interpretation of these results is that the trends reflect the ability of reduced protein to slow many aspects of the aging process and reduced dietary yeast to extend lifespan [26], [36], [37]. However, given the observation that the low-yeast diet does not simply slow age-dependent changes in total CHC levels but eliminates them entirely, it is more likely that production of some CHCs, particularly those that experience increased synthesis with age, is stimulated by dietary protein, or possibly, by other compounds in the yeast. Whether these CHCs have defined functions and dietary yeast is required for their synthesis or whether these CHCs are just by-products of changes in other metabolic pathways remain open questions.

**Figure 2:**
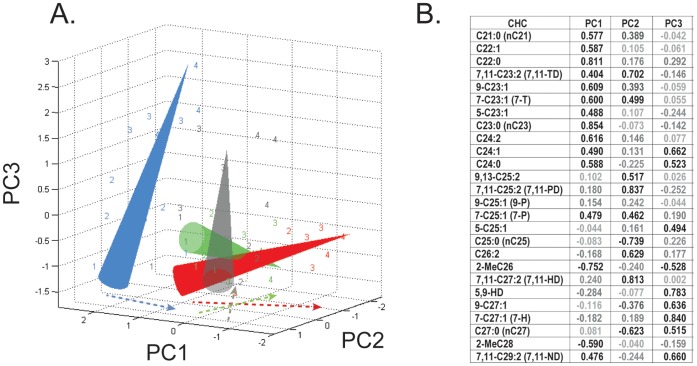
Principal component analysis of CHCs detected by GC-MS in *D. melanogaster* females fed four different diets. Three PCs were retained that explained 56% total variance, with PC1, PC2, and PC3 explaining 22%, 18%, and 16% of variance after Varimax rotation. PC scores are plotted in 3D space (A), and orthogonal best fit lines for PC scores serve as axes of cones with apexes pointing in the direction of increasing fly age. The numbers designate fly ages: 1 = 7d, 2 = 23d, 3 = 49d, 4 = 65d. Colors indicate food treatments: Grey = S5Y5, Red = S5Y20, Blue = S20Y5, Green = S20Y20. The arrows on PC1-PC2 plane represent the projections of each cone’s axis. PC loadings are shown in the table (B) with shading reflecting the strength of each CHC’s load on each PC (as rendered by JMP). For statistical treatment of individual CHCs, see Fig. S2.

To gain insight into the global response of CHC profiles to diet, we first visualized relative amounts of individual CHCs using principal component analysis. For 26 CHCs identified by GC-MS analysis, three retained PCs explained 56% of all variation (Fig. 2, Fig. S1). To compare the age-dependent changes in each of the diets, we plotted PC scores in 3D space and identified the major trends with an “aging cones”. These cones resulted from fitting an orthogonal regression line through all samples of the same diet treatment, with the base of the cone representing the youngest ages. The plot provides several useful insights. First, it illustrates that females fed the S20Y5 diet exhibited CHC profiles that were clearly distinct from the profiles measured on females from the other diets (Fig. 2A, focus on the blue cone). Second, the differences among the diets increased with age (i.e., the Euclidean distance between the cones increased towards their tips). Third, the two unbalanced diets, S20Y5 and S5Y20, diverged most dramatically (Fig. 2A, compare red and blue cones), and the balanced diets of S5Y5 and S20Y20 (as well as S10Y10 in Fig. S1) affected CHCs in an intermediate fashion between sugar- and yeast-biased diets. Fourth, the S20Y20 cone more closely followed changes induced by S5Y20 diet (Fig. 2A, the green cone is closest to red), which indicates a dominance of yeast effects over sugar. Similarities between S20Y20 and S5Y20 can be partly explained by the increase in total CHCs on high-yeast diets (Fig. 1). However, changes in total CHC levels cannot explain all of the observed responses to diet because the CHC profiles of S5Y5 females (Fig. 2A, grey cone) and of S20Y5 females are significantly different (Fig. 2A, blue cone) despite exhibiting similar total CHC amounts (Fig. 1).

Some interpretation of the meaning of each PC in terms of the original CHCs can be provided by examining the PC loadings (Fig. 2B). Thus, PC1 is characterized by positively loading short-chain CHCs (C21–24) and by negatively loading Me-group CHCs. Females fed a S20Y5 diet (Fig. 2A, blue cone) have high PC1 values, which decline slowly with age, while females fed a S5Y20 diet (Fig. 2A, red cone) have lower PC1 values early in life that decline rapidly in an age-dependent manner. PC2 is represented primarily by medium-long-chain CHCs, and it includes positively loading dienes 7,11-TD, 9,13–C25∶2, 7,11-PD, C26∶2, and 7,11-HD (the major identified female sex pheromone) and negatively loading alkanes C25∶0 and C27∶0. PC2 scores for all diets except S20Y5 decrease with age. PC3 is represented by a number of mostly long-chain CHCs, including 5,9-HD, 9–C27∶1, 7-H, and 7,11-ND, and it distinguishes high-yeast diets (S5Y20 and S20Y20) from low-yeast diets (S5Y5 and S20Y5). For flies from high-yeast diets, PC3 scores are not significantly different at different ages, while they increase with age for flies from low-yeast diets (Fig. 2A).

**Figure 3:**
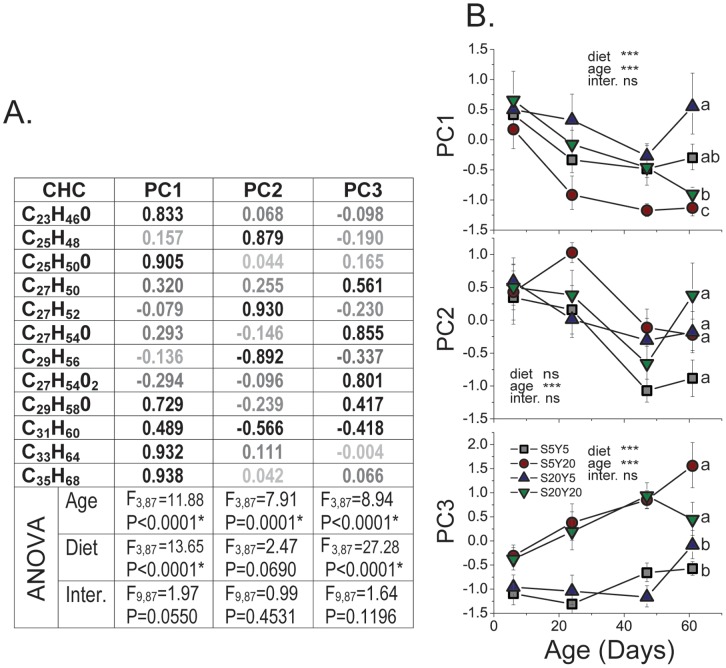
Principal component analysis of CHCs detected by LDI-MS in *D. melanogaster* females fed four different diets. Three retained PCs explain 80% of the total variation in CHCs, with PC1, PC2, and PC3 explaining 36%, 24%, and 19% of variance respectively after Varimax rotation. Table (A) shows PC loadings. ANOVA analyses were performed on log-transformed component scores for rotated PCs. Three PC scores are each plotted against female age of 7, 28, 43, and 58 days (B). Colors and symbols indicate food treatments: Grey squares = S5Y5, Red circles = S5Y20, Blue triangles = S20Y5, Green inverted triangles = S20Y20. Abbreviations used: ns = not significant (at α<0.05), inter. = interaction effect. Letters on the right, where different, specify statistically significant difference (at α<0.05) between diets by Tukey HSD tests. For statistical treatment of individual CHCs, see Fig. S3.

PCA was also applied to a dataset comprised of the 12 CHCs identified by LDI-MS analysis [38]. The three retained PCs explained 80% of the total CHC variation, and the PC structure was easily interpretable–most individual CHCs loaded heavily on one of the PCs (Fig. 3A). PC1 is strongly represented by three O-containing compounds, C_23_H_46_O, C_25_H_50_O, C_29_H_58_O, and two very long-chain CHCs, C_33_H_64_ and C_35_H_68_. S5Y20 females exhibited the lowest value for PC1, while females from the S20Y5 diet had the highest PC1 values. Statistically, PC1 effectively separates females fed a S5Y20 diet from the remaining three diet treatments (Fig. 3B, top panel). Very long-chain CHCs (C31–35) are prevalent in young flies, but decreased significantly within few days after eclosion [17], suggesting that their further loss in aging females fed S5Y20 may reflect accelerated senescence induced by the unbalanced and protein-rich nature of the diet (Fig. 3B, red line in top panel). PC2 (Fig. 3B, middle panel) is represented by positively loading C_25_H_48_ (presumably, 7,11-PD) and C_27_H_52_ (7,11-HD) and by negatively loading C_29_H_56_ (7,11-ND). This PC was not particularly associated with any one diet, but its scores universally decreased with age. Two Oxygen-containing compounds, C_27_H_54_O and C_27_H_54_O_2_, load heavily on PC3, and this PC clearly distinguishes females fed high- and low-yeast diets (Fig. 3B, bottom panel). Several insects have been shown to metabolize CHCs by hydroxylation and oxidation into O-containing compounds that serve as contact pheromones [39]. The functions of Oxygen-containing CHCs in *D. melanogaster* females, however, are unknown (but see [40] for possible functions in males).

In summary, the PCA analysis suggests several global trends. First, most individual CHCs can be assigned to one of several groups, each of which responded characteristically to diet. Second, the most dramatic differences in CHC profiles were between sugar- and protein-rich diets. Third, diet-mediated differences in CHCs profiles increased with age. Fourth, dietary yeast tended to exert dominant effects over sugar on the relative abundances of individual CHCs as well as on total CHC amount.

### Contrasting Effects of Dietary Yeast and Sugar on Individual CHCs

**Figure 4:**
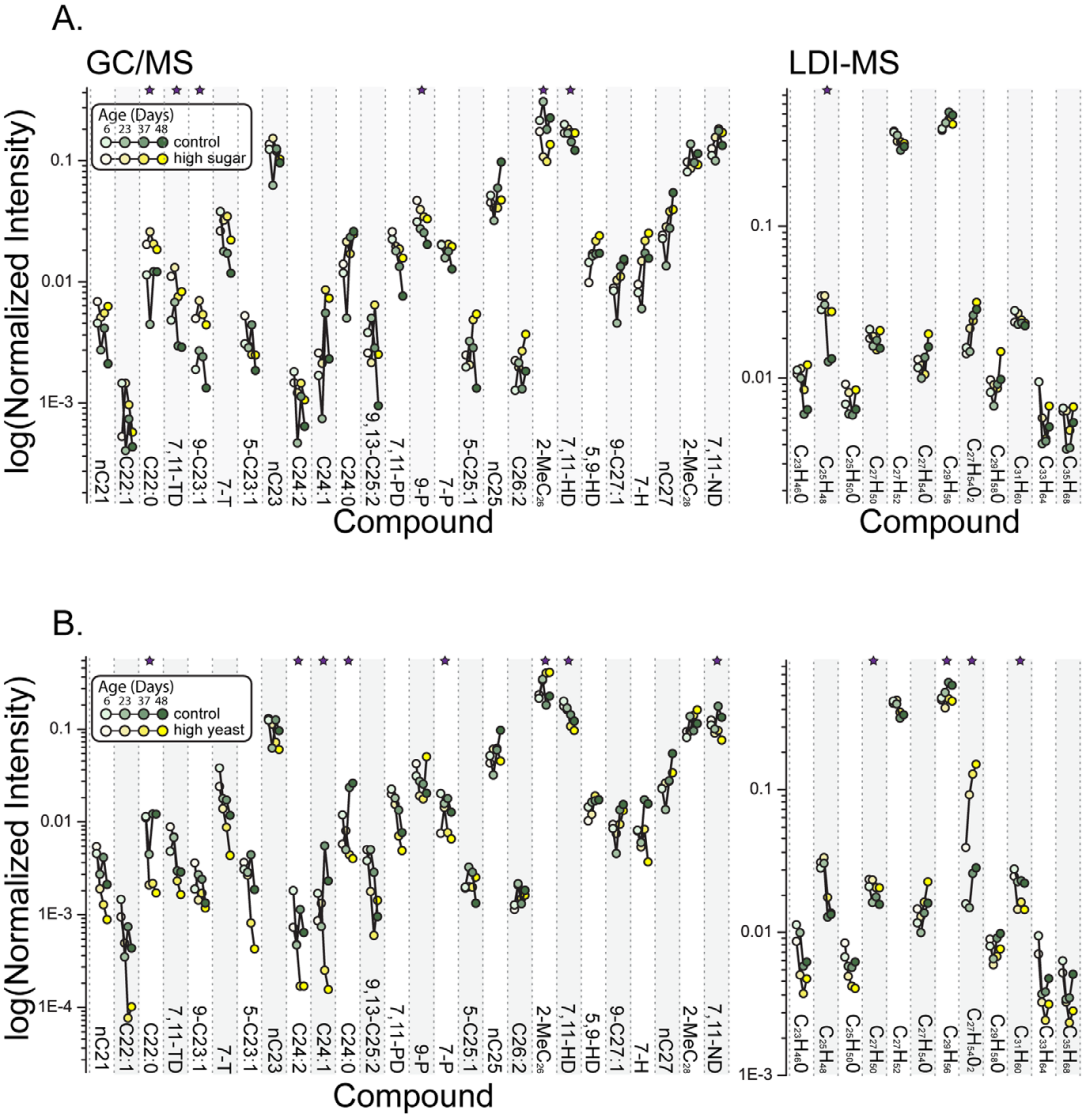
Effects of dietary sugar and yeast on *D. melanogaster* female CHCs. Normalized intensities of individual CHCs are shown across 4 ages (7, 23, 49, and 65 days) for females fed balanced S5Y5 diet (control) as opposed to high-sugar S20Y5 diet (A) or high-protein S5Y20 diet (B). Stars indicate significant effect based on 2-factorial ANOVA models of diet and/or diet by age interaction after sequential Bonferroni correction.

Examination of diet- and age-dependent changes in individual CHCs reveals a number of interesting trends. Consistent with our interpretation of the PCA, we found that dietary yeast and sugar produced contrasting effects on many individual CHCs (Fig. 4A and B, note the log-scale on the Y axes). Following correction for multiple testing, several CHCs, namely C22∶0, 7,11-TD, 9–C23∶1, 9-P, 7,11-HD, and C_25_H_48_ significantly increased in relative abundance following an increase in dietary sugar (S5Y5 vs. S20Y5; Fig. 4A). These initial differences persisted throughout the lifespan. On the other hand, the relative abundances of several CHCs were reduced in females fed high-yeast diets (S5Y5 vs. S5Y20); 8/26 CHCs from the GC-MS dataset and 4/12 CHCs from the LDI-MS dataset were significantly decreased, with effects often magnified by aging. Unlike most other CHCs, a methyl-group compound, 2-MeC_26_, that constitutes between 10–40% of the total amount of CHCs detected by GC-MS, experienced a roughly two-fold increase in its relative abundance with age on the high-yeast (S5Y20) diet. Levels of this compound were significantly reduced and independent of age in females fed a sugar-biased diet (Fig. 4B, Fig. S2). These data complement a growing body of literature suggesting that dietary yeast and sugar have opposing effects on a range of phenotypes including triglyceride levels, fecundity, and sleep/activity patterns [25], [26]. It seems likely that this represents a general phenomenon.

LDI-MS analysis revealed that increased dietary yeast resulted in a significant upregulation of the oxygen-containing compound C_27_H_54_O_2_, which increased 3-fold with age on S5Y20 diet (Fig. 4B and Fig. S3). This compound may, therefore, serve as an indicator of the amount of protein or lipid in female diet, the former of which is known to correlate strongly with fecundity in mated females [26]. LDI-MS analysis was generally consistent with the GC-MS results for those compounds that were detected with both methods (e.g. C_27_H_52_ = 7,11-HD, and C_29_H_56_ = 7,11-ND).

In addition to the main effects of dietary sugar and yeast, our experimental design provides the ability to determine the effects of balanced versus unbalanced diets and to assess specific CHCs for which the dietary components exhibit dominance or additivity in their effects. For the majority of CHCs analyzed using GC-MS, relative abundances on balanced diets were roughly intermediate to the relative abundances observed on yeast- and sugar-rich diets (i.e., grey lines/square symbols and green lines/inverted triangle symbols lie close to each other and between red lines/round symbols and bluelines/triangle symbol, Fig. S2). For these compounds, it appears that individual dietary components exert independent/additive effects on CHC profiles, perhaps by altering substrate availability for different CHC synthesis pathways (see below). On the other hand, the S20Y20 diet often resulted in relative CHC abundances that were more similar to those observed in females from the yeast-rich (5S/20Y) diet than they were to females from the sugar-rich (S20Y5) diet (green line is closer to the red than to the blue line in Fig. S2); these compounds are regulated predominantly by dietary yeast.

**Figure 5:**
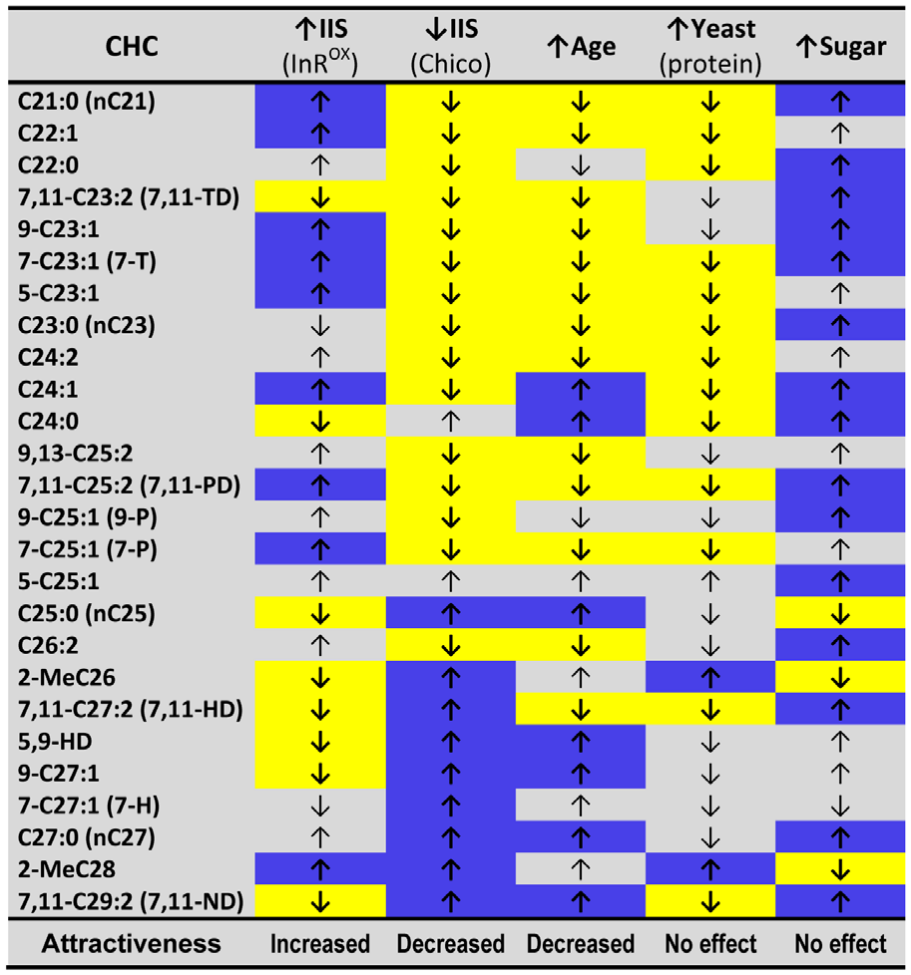
Comparison of diet, age, and insulin signaling effects on CHCs. Arrows indicate an increase or decrease in the relative abundance of individual CHCs: 1) for aging - from young to old age on balanced diets [18], 2) for protein – based on change from S5Y5 to S5Y20, and for sugar – based on change from S5Y5 to S20Y5, 3) for insulin signaling (IIS) - change in transgenic flies with increased insulin signaling (via insulin receptor overexpression - InR^OX^) or decreased insulin signaling (insulin substrate mutants *chico*) compared to controls [19]. Yellow and blue background indicates statistically significant (at α = 0.05) decrease and increase, respectively. Our previous studies determined that increased insulin signaling or young age makes females more attractive to males and that decreased insulin signaling or old age makes them less attractive [14], [15].

Two pairs of CHCs – 2-MeC_26_ with 7,11-HD, and 2-MeC_28_ with 7,11-ND – stand out because of the similarity in molecular weight within each pair and because they are clearly the most abundant set of compounds detected by the GC-MS analysis [18]. These two pairs exhibited interesting responses to balanced and unbalanced diets. Within each pair, levels of the two CHCs exhibited opposing responses to yeast-rich and sugar-rich diets but similar responses to balanced diets of different nutritional value (e.g., S5Y5 *vs*. S20Y20; Fig. S4, see also [18], [19]). It has been shown that Me-branched and unsaturated CHCs are synthesized via two different pathways, both using the same substrate (Acetyl-CoA), and that 2Me-CHC synthesis utilizes several amino acids as its initial substrate [11], [39]. These two pathways may, therefore, compete for a common substrate, with their balance shifted in favor of the methyl-group compounds when dietary protein is replete and to the unsaturated CHC when it is limited (Fig. S4). Regardless of the mechanism, the relative representation of Me-branched CHCs compared to their unsaturated straight-chain CHC partners, 7,11-HD and 7,11-ND, seem to be a key trait that reflects the ratio of sugar versus yeast in the female diet. It will be important to determine whether Me-group compounds constitute attractive or repellent CHCs as well as whether they interact with 7,11-HD and 7,11-ND to determine overall female attractiveness. For instance in long-horned beetles, 2-MeC_26_ and 2-MeC_28_ have been shown to be the major female sex pheromones [41]. In *D. melanogaster*, however, the relative abundance of 2-MeC_26_ is increased (decreased) in females with decreased (increased) IIS, which are less (more) attractive to males (Fig. 5, see also [19]).

A comparison of how diet-dependent changes in CHC profiles correspond to those previously observed following aging and alteration of IIS may provide insights into the function of individual CHCs as well as the mechanisms of their regulation (Fig. 5). For example, the decreased relative abundance of short-chain CHCs that we observed in females fed a yeast-rich diet is also characteristic of old females kept on balanced diets [18] and of transgenic females with decreased IIS [19], suggesting that these physiological states may share some underlying regulatory mechanisms. Increased dietary protein may promote TOR signaling, which would result in increased protein synthesis and more rapid senescence [5], [42], [43]. Therefore, the decrease in short-chain CHCs in flies from high-yeast diets might reflect the idea that they are physiologically older than their siblings of the same chronological age fed low-yeast diets. Alternatively, ovarian activity may represent one of the important regulators of female CHC profiles, as has been shown for social insects [39]. Ovarian activity is low in aged females, females with decreased IIS, and females fed a low-yeast diet [44]. Therefore, compounds changed similarly by all these three conditions, such as increased levels of 7,11-ND, may indicate lower ovarian activity. Similarly, for short-chain CHCs, a high-sugar diet mimics the effects of increased insulin signaling (Fig. 5), which is consistent with modulation of this pathway by dietary carbohydrates [45].

While changes in short-chain CHC may be indicative of common regulatory mechanisms, there is less correspondence among changes in long-chain CHCs in response to age, diet, and IIS. For example, while the changes in shorter-chain (C21–26) and Me-branched CHCs in response to increased dietary yeast are similar to those induced by decreased IIS and aging, changes in the levels of long-chain dienes (7,11-HD and 7,11-ND) to the same diet mimic increased IIS (Fig. 5). Similarly, for sugar-rich diets changes in shorter-chain CHCs (C21–26) and 2Me-C_26_ phenocopy those caused by increased IIS, while changes in 7,11-HD and 7,11-ND phenocopy decreased insulin signaling. Interestingly, increased dietary sugar has been shown to inhibit elongation and promote desaturation of fatty acids in *Drosophila* larvae [46].

### Effect of Diet on Female Attractiveness

**Figure 6:**
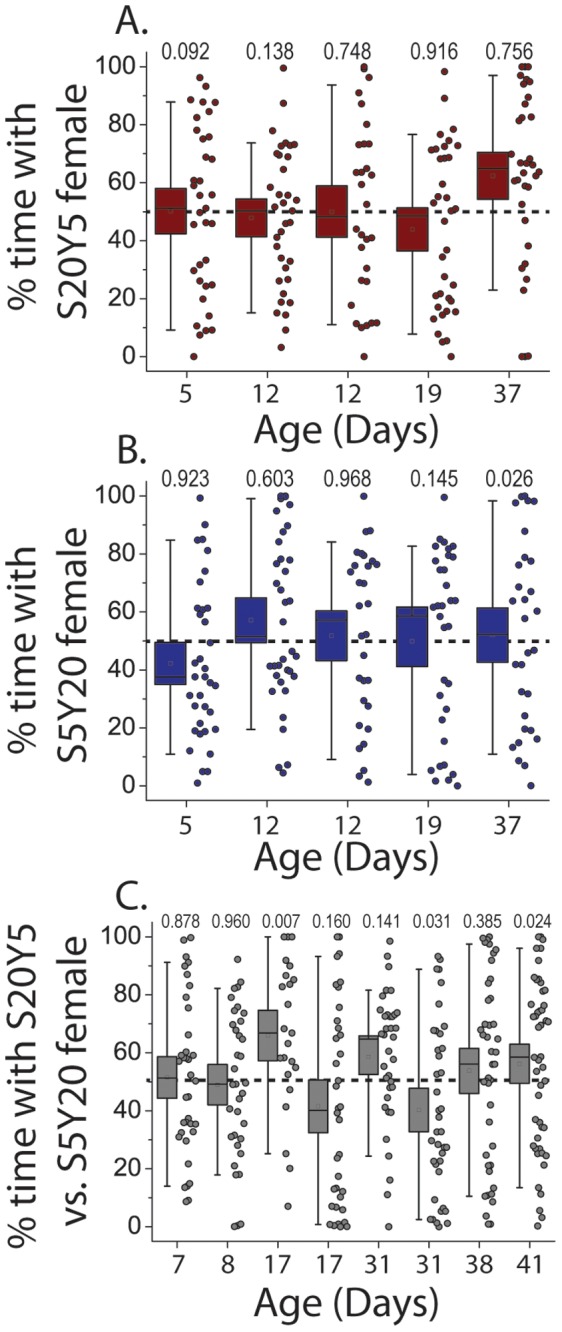
Effect of diet on female attractiveness. Females fed the two unbalanced diets (S20Y5 and S5Y20) are not different in attractiveness when tested against S5Y5 females (A,B), or against each other (C). Female attractiveness is expressed as % time a male spends in courtship proximity of a target female in 2-choice assays. P-values are shown above corresponding box plots. While few replicates show a difference from 50% (dotted line), these are not consistent in direction and not significant after Bonferroni correction.

We have shown previously that the changes in CHC profiles induced by aging and by manipulations of IIS impact sexual attractiveness [18], [19]. In those studies, males exhibited a 70–80% preference towards young (vs. old) females and a 60–75% preference for transgenic females with higher IIS. In the current study, we found that diet significantly impacts CHC profiles, suggesting that it might affect attractiveness as well. Surprisingly, we obtained no evidence for significant dietary effects on attractiveness. Females fed yeast-rich (S5Y20) or sugar-rich diets (S20Y5) did not consistently differ in attractiveness to males when tested against S5Y5 females (Fig. 6A,B). Because females fed the two unbalanced diets exhibited the largest difference in their CHC profiles, they were also placed in competition with one another in our choice assay. However, we failed to observe consistent differences over measures spanning most of female lifetime (Fig. 6C). A few trials indicated modest statistically significant differences between dietary treatments, but the direction of the effect varied from trial to trial and none remained significant after a Bonferonni correction for multiple testing. Taken together these data indicate that the broad range of dietary conditions that was used in these experiments does not affect female attractiveness.

Interestingly, a positive effect of sugar (or negative effect of yeast) on female attractiveness has been reported for *D. melanogaster* fed sugar-only (S10) or S10Y10 diets [47]. Despite using similar conditions, however, we failed to observe differences in attractiveness between females fed sugar-only diet and females fed a balanced S10Y10 diet (Fig. S6). This inconsistency may result from differences in fly husbandry or in experimental conditions or protocols. For instance, Cook and Cook [47] used decapitated females that were not fixed in place, which allowed them to express a limited range of normal behaviors (such as preening or basic responses to male courtship; *personal observation*). Our protocol requires the females’ legs to be fixed in agar, and we have shown that this behavioral assay results in male choice based on CHC perception and not on other female traits or behaviors [18], [19].

Finally, combined analysis of changes in attractiveness in response to diet, aging, and IIS, indicates that attractiveness is likely to be determined by a combination of several attractive and repulsive CHCs. Previous studies suggested that it is female unsaturated CHCs (especially female-specific dienes), and, in particular 7,11-HD, that are biologically active and that alkanes do not induce male courtship [14], [15]. Therefore, we asked whether diet composition is comparable to aging and IIS manipulation in its effects on the prevalence of major CHC classes, such as those defined by their carbon chain length or the number of double bonds or Me-group present. While all these manipulations seem to affect the proportion of the four major CHC classes, there is no pattern consistent with effects on attractiveness (Fig. S5A). Similar to what we observed previously [18], [19], however, we did observe a noticeable trend in the effects of diet on CHCs of different carbon chain length (Fig. S5B). In the yeast-rich diet we observed an age-dependent increase in the representation of long-chain CHCs and a corresponding decrease in short-chain CHCs, as evidenced by significant age*chain-length interaction. The sugar-rich diet resulted in an increased prevalence of short-chain CHCs and a decrease in long-chain CHCs, which was largely unchanged with age (Fig. S5B). However, this similarity in the effect of diet and age on CHC chain length did not result in similar effect of these manipulations on attractiveness, suggesting that other characteristics of CHC profiles must be important. In addition, the lack of correlation between attractiveness and relative abundance of 7,11-HD in females with altered IIS [19] suggests that other CHCs must influence attractiveness, perhaps by masking or opposing the effect of 7,11-HD. Notably, changes in 2-MeC_26_ and 2-MeC_28_ on unbalanced diets of variable quality are accompanied by opposing changes in 7,11-HD and 7,11-ND, while aging and reduced IIS on balanced diets each lead to positively correlated changes in these CHCs.

We should note that shorter-chain CHCs respond similarly to increased dietary yeast, aging, and decreased IIS, but long-chain CHCs respond quite differently across these treatments (Fig. 5). It is possible, therefore, that changes in individual CHCs work in opposition, effectively cancelling each other out, and leaving no overall effect on attractiveness. In other words, it may be that the benefits of increased levels of certain attractive CHCs may be limited by reductions in other attractive CHC or increases in repulsive CHCs.

### Conclusions

This study found consistent and significant effects of diet composition on female CHC profiles. PCA analyses and comparison of CHC levels across a range of different diets suggested that 1) most individual CHCs tend to cluster into a small number of groups, each of which respond to diet differently; 2) dietary sugar and yeast generate opposite changes in CHC profiles; 3) yeast effects are generally dominant over those of sugar; and 4) the magnitude of diet-dependent effects is increased with age. A simple meta-analysis of diet-induced changes in CHCs together with those caused by aging and IIS [18], [19] provide potentially valuable insight into the function of individual compounds and their underlying regulatory mechanism. Our analysis suggests, for example, that responses of short-chain CHCs to diet might be mediated by the IIS pathway, while long-chain CHCs might have a separate regulatory mechanism.

Despite the profound changes in CHC profiles caused by different diets, these changes failed to lead to differences in male mating preference for females fed different diets. The lack of dietary effects on attractiveness is particularly surprising in the light of our previous data, which established that changes in CHCs profiles following aging and genetic manipulation of IIS strongly affect female attractiveness [18], [19]. One potential explanation for these observations is that diet-related effects are complicated, and integrated over multiple, nutrient-dependent molecular pathways. This may lead to changes in some CHCs that reduce attractiveness and others that potentiate it, thereby sending conflicting messages to potential mates. In support of this hypothesis, changes in short-chain and Me-branched CHCs in response to increased dietary protein phenocopy changes with aging and decreased IIS, while changes in the long-chain CHCs–specifically the most abundant compounds 7,11-HD, and 7,11-ND–do not exhibit similar trends. In fact, these often change in the opposite direction from that predicted based on changes in short-chain CHCs.

Our results have several implications for CHC biology and evolutionary theory. First, we have identified several compounds, including predominant Me-branched and oxygen-containing CHCs, that respond differentially to diet. These CHCs deserve further investigation as potential attractive or repulsive pheromones in *Drosophila*. Second, in terms of evolutionary theory, it is known that dietary changes in natural and laboratory insect populations at both larval and adult stages often lead to changes in CHC composition, nestmate recognition, and mate preferences [10], [48], [49], [50]. In the present study, we have not observed consistent effects of diet-induced changes in CHCs on female attractiveness, suggesting that not all dietary-induced CHC changes generate differences in attractiveness. Finally, the experimental and analytical approaches used in this and our previous studies, which include genetic and environmental manipulations that break down correlations among individual and groups of CHCs, followed by multivariate analysis of these changes, may be valuable for identifying CHCs that are important for attractiveness, and at the same time, for determining the underlying biosynthetic pathways responsible for the observed CHC changes. Together, these considerations may clarify why certain CHCs are used in mate choice. CHCs that define attractiveness may be the very ones that accurately reflect the activity of the evolutionarily conserved molecular pathways that are critical determinants of reproductive success and overall fitness [19].

## Supporting Information

Figure S1 **Principal component analysis of CHCs detected by GC-MS in** ***D. melanogaster*** **females fed different diets.** The plot represents the same data as in Fig. 2 with the addition of two more aging cones for Canton-S (purple) and a second wild type strain called Fv (yellow), which we used to increase the power of the PCA analysis. Both strains were fed S10Y10 diet; additional details are presented elsewhere [18]. The similar directions of the purple and yellow cones in 3D space indicate that genetic background has minimal effect on CHC aging dynamics and that the measures are highly repeatable. The intermediate position of these two cones between the balanced treatments of S5Y5 (grey) and S20Y20 (green) suggests a dose-dependent effect of diet.
(TIF)

Figure S2 **Aging dynamics of individual CHCs identified with GC-MS analysis in** ***D. melanogaster*** **females fed four different diets**. X-axis indicates the age of females (7, 23, 49, and 65 days). P-values for the effects of diet, age, and diet by age interaction are coded as *(P<0.05), **(P<0.01), ***(P<0.001), and ns (P>0.05). Colored symbols indicate food treatments: Grey squares = S5Y5, Red circles = S5Y20, Blue up-triangles = S20Y5, Green down-triangles = S20Y20, and lower-case letters on the right, where different, indicate statistically significant difference (at α<0.05) between diets by Tukey HSD tests.
(TIF)

Figure S3 **Aging dynamics of individual CHCs identified with LDI-MS analysis in** ***D. melanogaster*** **females fed four different diets**. X-axis indicates the age of females (7, 28, 43, and 58 days). P-values for the effects of diet, age, and diet by age interaction are coded as *(P<0.05), **(P<0.01), ***(P<0.001), and ns (P>0.05). Colored symbols indicate food treatments: Grey squares = S5Y5, Red circles = S5Y20, Blue up-triangles = S20Y5, Green down-triangles = S20Y20, and letters, where different, specify statistically significant difference (at α<0.05) between diets by Tukey HSD tests.
(TIF)

Figure S4 **Relationships between relative abundance of 2Me-C_26_ and 7,11-HD on different diets.** There is a significant negative correlation between the relative abundances of these compounds, which may reflect competing substrate use by the two biosynthetic pathways for dienes and Me-group CHCs. The numbers designate fly ages: 1 = 7d, 2 = 23d, 3 = 49d, 4 = 65d, and colors indicate diet treatment (Grey = S5Y5, Red = S5Y20, Blue = S20Y5, Green = S20Y20).
(TIF)

Figure S5 **Effects of diet on major CHC classes and chain length:** (**A**) A proportional representation of the major CHC classes (alkanes, monoenes, dienes, and Me-alkanes) in CHC profiles of *D. melanogster* females in response to differences in (left to right): reduced insulin signaling through mutation of *chico*; increased insulin signaling through overexpression of the insulin receptor, *InR*; aging (7 *vs* 49 day old flies); diets rich in either sugar or protein. (**B**) Change in the relative abundance of individual CHCs of variable chain length in response to high-sugar (S20Y5 minus S5Y5) or high-yeast (S5Y20 minus S5Y5) diets. P-values associated with regression analysis of % change on carbon chain length are presented in each age panel. A multiple regression analysis indicates significant effects of age (P = 0.006), chain length (P = 0.02), and their interaction (P = 0.03) on the change in the proportions of individual CHCs in response to high-protein diet. High-sugar diet significantly altered CHC chain length (P = 0.0003), but neither age (P = 0.31) nor the interaction between age and chain length (P = 0.96) were statistically significant.
(TIF)

Figure S6 **Effect of regular S10Y10 diet versus sugar-only (S10) diet on female attractiveness**. No consistent male preference was observed for 4–6 day old females fed either diet competed against each other in the two-choice attractiveness assay. Three replicate experiments were performed, and P-values indicate difference from 50% based on Wilcoxon signed rank test.
(TIF)
