# Supplementary figures and images for: Dietary Effects on Cuticular Hydrocarbons and Sexual Attractiveness in Drosophila
Source: PLoS One. 2012 Dec 5;7(12):e49799. doi: 10.1371/journal.pone.0049799 (PMC3515564; doi:10.1371/journal.pone.0049799)

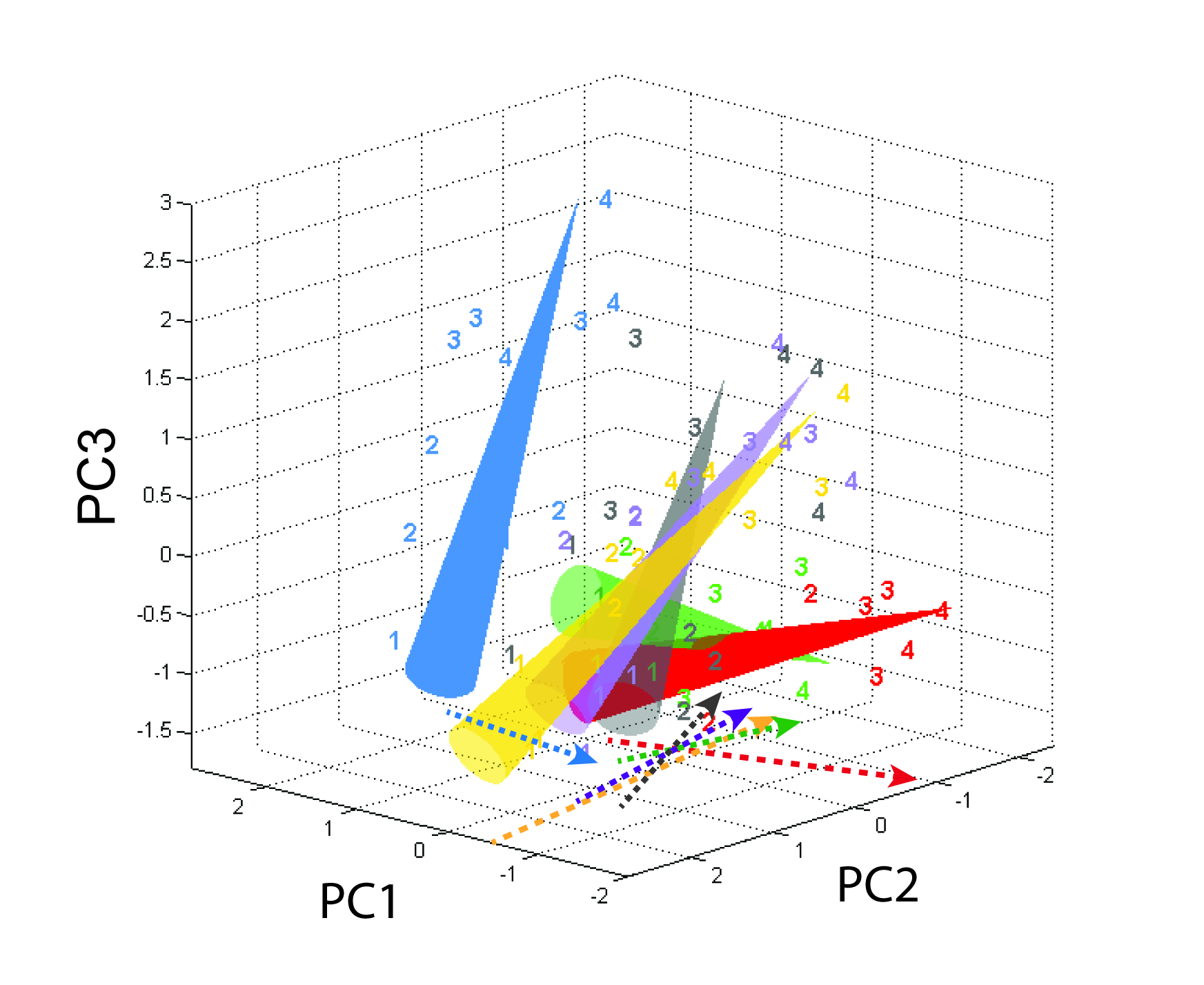

Supplement: Figure S1 — Principal component analysis of CHCs detected by GC-MS in D. melanogaster females fed different diets. The plot represents the same data as in Fig. 2 with the addition of two more aging cones for Canton-S (purple) and a second wild type strain called Fv (yellow), which we used to increase the power of the PCA analysis. Both strains were fed S10Y10 diet; additional details are presented elsewhere [18]. The similar directions of the purple and yellow cones in 3D space indicate that genetic background has minimal effect on CHC aging dynamics and that the measures are highly repeatable. The intermediate position of these two cones between the balanced treatments of S5Y5 (grey) and S20Y20 (green) suggests a dose-dependent effect of diet. (TIF) [file pone.0049799.s001.tif]

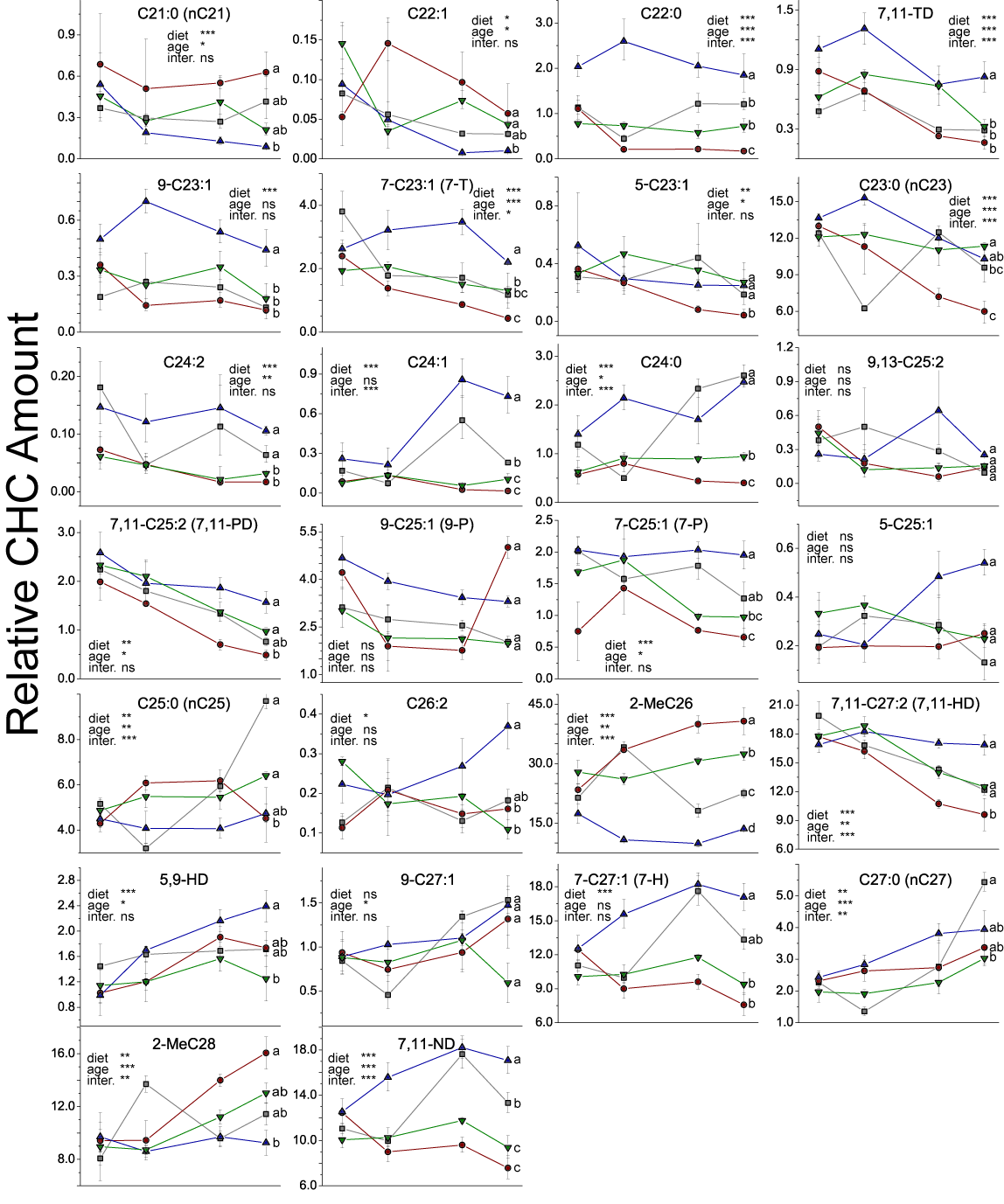

Supplement: Figure S2 — Aging dynamics of individual CHCs identified with GC-MS analysis in D. melanogaster females fed four different diets. X-axis indicates the age of females (7, 23, 49, and 65 days). P-values for the effects of diet, age, and diet by age interaction are coded as *(P<0.05), **(P<0.01), ***(P<0.001), and ns (P>0.05). Colored symbols indicate food treatments: Grey squares = S5Y5, Red circles = S5Y20, Blue up-triangles = S20Y5, Green down-triangles = S20Y20, and lower-case letters on the right, where different, indicate statistically significant difference (at α<0.05) between diets by Tukey HSD tests. (TIF) [file pone.0049799.s002.tif]

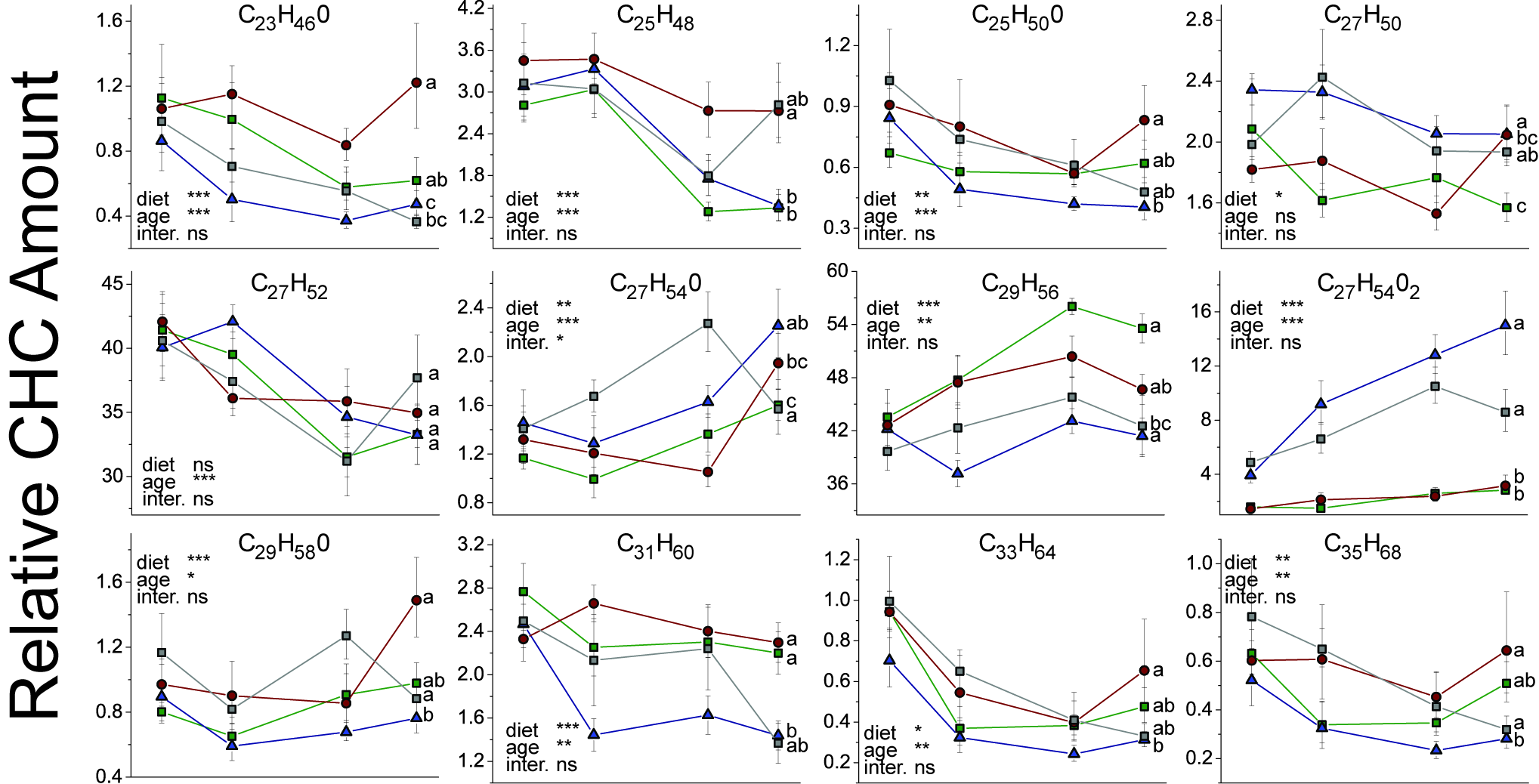

Supplement: Figure S3 — Aging dynamics of individual CHCs identified with LDI-MS analysis in D. melanogaster females fed four different diets. X-axis indicates the age of females (7, 28, 43, and 58 days). P-values for the effects of diet, age, and diet by age interaction are coded as *(P<0.05), **(P<0.01), ***(P<0.001), and ns (P>0.05). Colored symbols indicate food treatments: Grey squares = S5Y5, Red circles = S5Y20, Blue up-triangles = S20Y5, Green down-triangles = S20Y20, and letters, where different, specify statistically significant difference (at α<0.05) between diets by Tukey HSD tests. (TIF) [file pone.0049799.s003.tif]

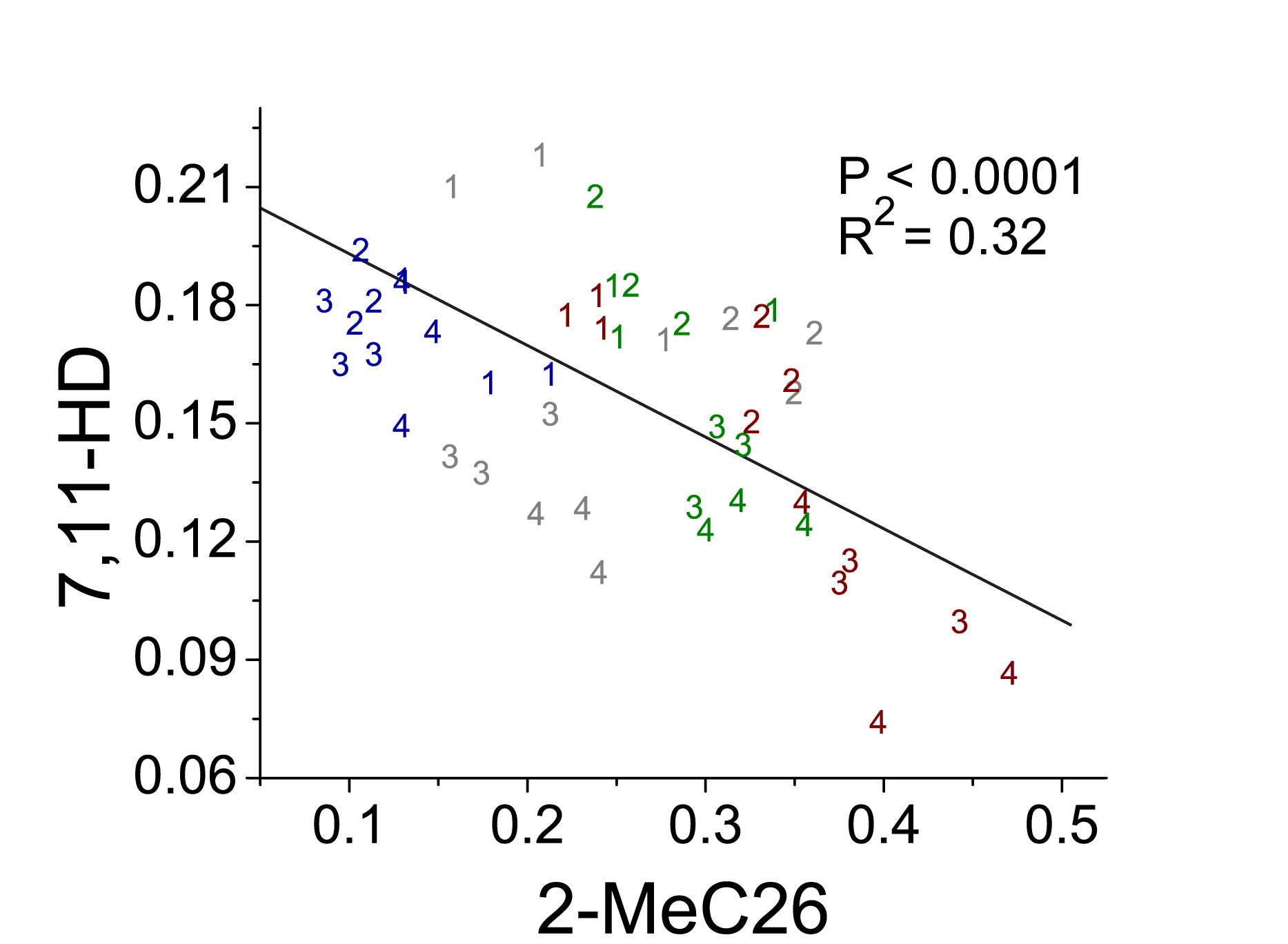

Supplement: Figure S4 — Relationships between relative abundance of 2Me-C26 and 7,11-HD on different diets. There is a significant negative correlation between the relative abundances of these compounds, which may reflect competing substrate use by the two biosynthetic pathways for dienes and Me-group CHCs. The numbers designate fly ages: 1 = 7d, 2 = 23d, 3 = 49d, 4 = 65d, and colors indicate diet treatment (Grey = S5Y5, Red = S5Y20, Blue = S20Y5, Green = S20Y20). (TIF) [file pone.0049799.s004.tif]

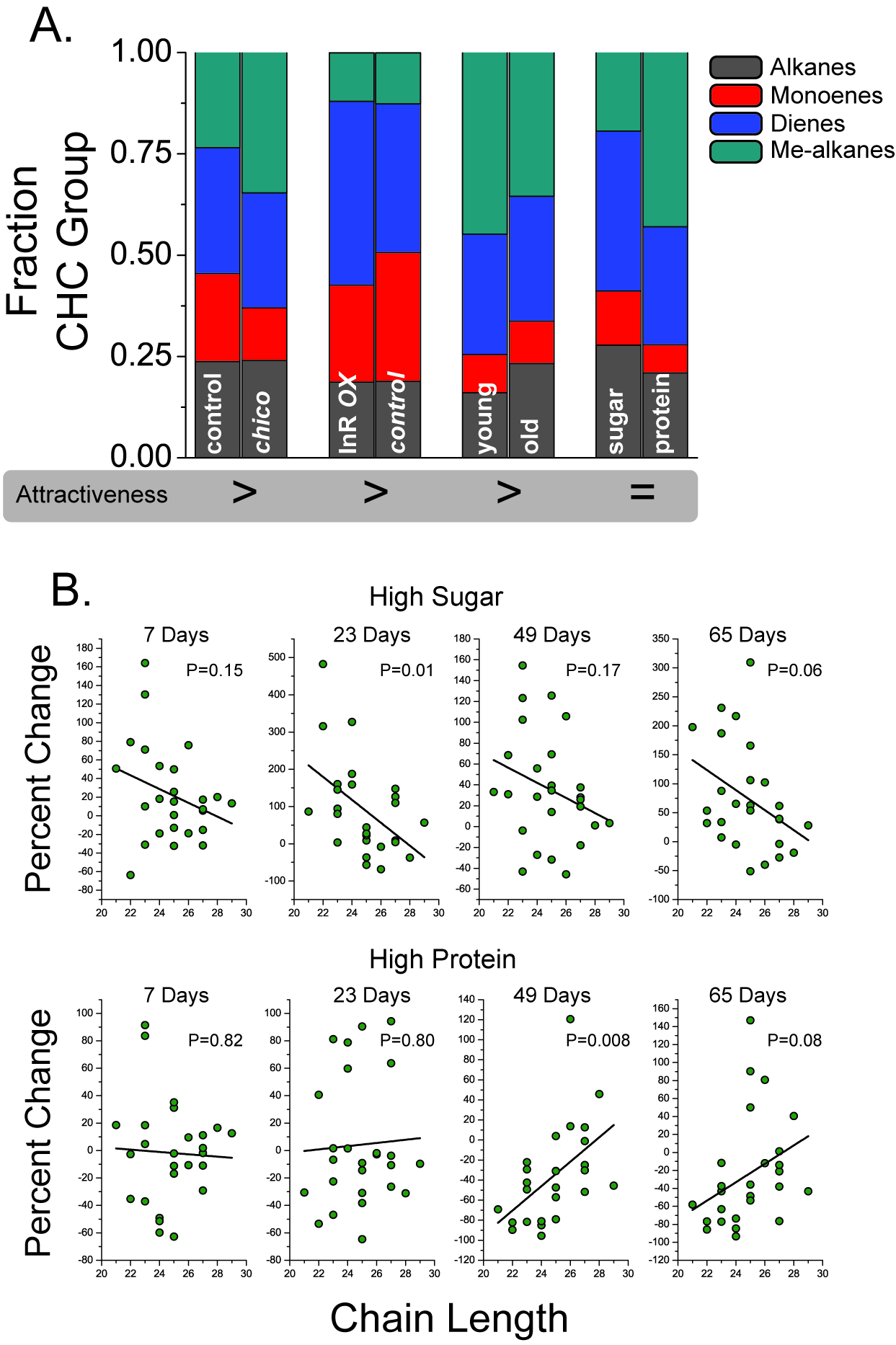

Supplement: Figure S5 — Effects of diet on major CHC classes and chain length: (A) A proportional representation of the major CHC classes (alkanes, monoenes, dienes, and Me-alkanes) in CHC profiles of D. melanogster females in response to differences in (left to right): reduced insulin signaling through mutation of chico; increased insulin signaling through overexpression of the insulin receptor, InR; aging (7 vs 49 day old flies); diets rich in either sugar or protein. (B) Change in the relative abundance of individual CHCs of variable chain length in response to high-sugar (S20Y5 minus S5Y5) or high-yeast (S5Y20 minus S5Y5) diets. P-values associated with regression analysis of % change on carbon chain length are presented in each age panel. A multiple regression analysis indicates significant effects of age (P = 0.006), chain length (P = 0.02), and their interaction (P = 0.03) on the change in the proportions of individual CHCs in response to high-protein diet. High-sugar diet significantly altered CHC chain length (P = 0.0003), but neither age (P = 0.31) nor the interaction between age and chain length (P = 0.96) were statistically significant. (TIF) [file pone.0049799.s005.tif]

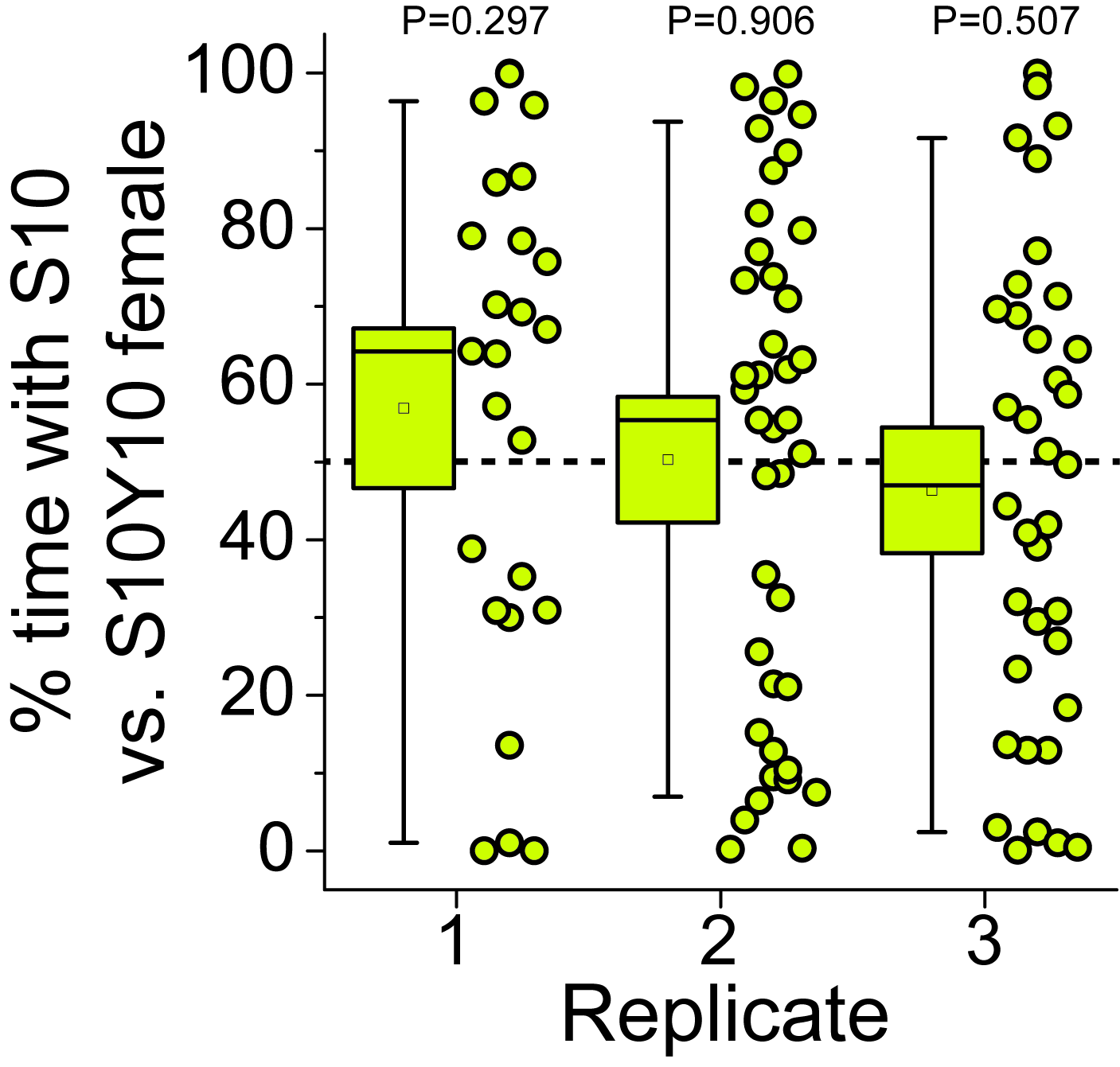

Supplement: Figure S6 — Effect of regular S10Y10 diet versus sugar-only (S10) diet on female attractiveness. No consistent male preference was observed for 4–6 day old females fed either diet competed against each other in the two-choice attractiveness assay. Three replicate experiments were performed, and P-values indicate difference from 50% based on Wilcoxon signed rank test. (TIF) [file pone.0049799.s006.tif]
